# *Allovahlkampfia spelaea* Causing Keratitis in Humans

**DOI:** 10.1371/journal.pntd.0004841

**Published:** 2016-07-14

**Authors:** Mohammed Essa Marghany Tolba, Enas Abdelhameed Mahmoud Huseein, Haiam Mohamed Mahmoud Farrag, Hanan El Deek Mohamed, Seiki Kobayashi, Jun Suzuki, Tarek Ahmed Mohamed Ali, Sumio Sugano

**Affiliations:** 1 Department of Parasitology, Faculty of Medicine, Assiut University, Assiut, Egypt; 2 Department of Medical Genomics, Graduate School of Frontier Sciences, The University of Tokyo, Minato-ku, Tokyo, Japan; 3 Faculty of Applied Medical Science, Shaqra University, Shaqra, Saudi Arabia; 4 Department of Infectious Diseases, Keio University School of Medicine, Shinjuku-ku, Tokyo, Japan; 5 Division of Clinical Microbiology, Department of Microbiology, Tokyo Metropolitan Institute of Public Health, Shinjuku-ku, Tokyo, Japan; 6 Department of Ophthalmology, Faculty of Medicine, Assiut University, Assiut, Egypt; Jawaharlal Nehru University, INDIA

## Abstract

**Background:**

Free-living amoebae are present worldwide. They can survive in different environment causing human diseases in some instances. *Acanthamoeba* sp. is known for causing sight-threatening keratitis in humans. Free-living amoeba keratitis is more common in developing countries. Amoebae of family *Vahlkampfiidae* are rarely reported to cause such affections. A new genus, *Allovahlkampfia spelaea* was recently identified from caves with no data about pathogenicity in humans. We tried to identify the causative free-living amoeba in a case of keratitis in an Egyptian patient using morphological and molecular techniques.

**Methods:**

Pathogenic amoebae were culture using monoxenic culture system. Identification through morphological features and 18S ribosomal RNA subunit DNA amplification and sequencing was done. Pathogenicity to laboratory rabbits and ability to produce keratitis were assessed experimentally.

**Results:**

*Allovahlkampfia spelaea* was identified as a cause of human keratitis. Whole sequence of 18S ribosomal subunit DNA was sequenced and assembled. The Egyptian strain was closely related to SK1 strain isolated in Slovenia. The ability to induce keratitis was confirmed using animal model.

**Conclusions:**

This the first time to report *Allovahlkampfia spelaea* as a human pathogen. Combining both molecular and morphological identification is critical to correctly diagnose amoebae causing keratitis in humans. Use of different pairs of primers and sequencing amplified DNA is needed to prevent misdiagnosis.

## Author Summary

Free-living amoebae are present worldwide. Some species are known to cause chronic keratitis in human. Amoebic chronic keratitis is sight-threatening disease occurring in both developing and well-developed countries. *Allovahlkampfia spelaea* is a newly discovered free-living amoeba. We report the first human case of chronic keratitis due to that amoeba. For correct identification, both morphological and molecular techniques should be combined.

## Introduction

Free-living amoebae (FLA) are present in different environments worldwide. They can survive in soil, surface water, other aquatic environments and even desert[1,2]. Some members of family Acanthamoebidae and Vahlkampfiidae are amphizoic, occurring as human parasite. The most commonly known genera are *Acanthamoeba* and *Naegleria* causing keratitis and primary amoebic meningoencephalitis[3,4]. Members of both families have vegetative form, the trophozoite, and quiescent form, the cyst, both can be used for morphological identification of different genera [5–7]. Cysts can survive for many years in environment as a potential source of infection[8].

Amoebic keratitis is an uncommon corneal disease that could eventually lead to loss of vision. It is usually associated with contact lens wearing or trauma[9,10]. The most common cause is the genus *Acanthamoeba* [4,11]. In India, *Acanthamoeba* keratitis was up to 2.5% of cases of non-viral keratitis and was more prevalent in rural poor areas[12,13]. *Vahlkampfia* was reported to cause keratitis with co-infection with *Acanthamoeba* or *Candida* [11,14,15]. Correct diagnosis is essential for treatment and prevention of vision loss. Diagnosis depends mainly on culture from corneal scraping [9,10] and molecular identification using polymerase chain reaction (PCR). Primers designed to amplify 18S ribosomal subunit are widely used[16–18].

In 2009, *Allovahlkampfia spelaea* was identified as a new genus and new species. It was reported as a FLA inhabiting cave in Slovenia [19]. Until now, there is no data about the ability of such amoeba to produce disease in human beings.

In this work, we aimed to identify FLA causing keratitis in an Egyptian patient using morphological and molecular approaches.

## Materials and Methods

### Parasite culture

Corneal scrapings from patient presented with keratitis were cultured on 1.5% non-nutrient agar made with Page’s saline and seeded with *Escherichia coli* kept in incubator at 30°C for 7 days. Cultures were examined using inverted microscope for presence of FLA every day and if FLA was detected, sub-culture was done every 10 to 14 days by inverting a slice on a new agar plate was done[9,20,21]. Morphology of trophozoites and cysts (non-stained and Giemsa’s Stained) was identified using light microscope and inverted microscope according to Smirnov and Goodkov (1999) and Smirnov and Brown (2004) [6,7]. Trial to axenize isolated FLA was done using Trypticase Soy Broth with Yeast Extract (TSY). Medium was prepared using BD Bacto Tryptic Soy broth (ref 211825) 30 grams, BD Bacto Yeast Extract (ref 212750) 10 grams and distilled water up to 1000 ml, pH adjust to 7.3 and autoclaved at 121°C for 15 minutes. To start axenic culture, cysts were collected using phosphate buffered saline (PBS) containing 0.01N HCL for 15 minutes, then washed 3 times in PBS by centrifugation at 600xg for 4 minutes. Cyst then were suspended in 10 ml TSY and kept in 25cm^2^ Falcon culture flasks (both vented and air tight flasks were tested).

### Molecular identification using 18S ribosomal subunit DNA

Cysts were collected and treated as previous in axenic trial. Cysts were monitored under inverted microscope for excystation and attachment of trophozoites to flask wall, then medium was decanted and replaced for 3 times to ensure only attached trophozoites were present. Preheated cell lysis solution (80°C) from Gentra Puregene Yeast/Bact. Kit B (Qiagen) was added to flask to ensure rapid lysis of trophozoites. Then DNA was extracted according to kit protocol.

### PCR amplification of 18S ribosomal subunit

We used 3 different pairs of primers to identify the FLA; JDP1 (5'GGCCCAGATCGTTTACCGTGAA) and JDP2 (5'TCTCACAAGCTGCTAGGGAGTCA)[16] as standard known primers to identify *Acanthamoeba*. Universal primers F-566 (5'CAG CAG CCG CGG TAA TTC C) and R-1200 (5' CCC GTG TTG AGT CAA ATT AAG C) as general primers for 18S ribosomal subunit [22], and Naeg-F (5'GAACCTGCGTAGGGATCATTT) and Naeg-R (5'TTTCTTTTCCTCCCCTTATTA) as general primers for ribosomal internal transcribed spacers (ITS) [3,20]. PCR reactions were done using Thermo Scientific Phusion High-Fidelity PCR Master Mix (ref F-531L) according to product manual for 35–40 cycles. PCR products were identified using 1% Agarose gel stained with ethidium bromide and were purified for sequencing using Qiaquick spin columns (Qiagen). Sequencing was done using Applied Biosystems 3730xl DNA Analyzer. Sequence data were retrieved and blasted using NCBI Blastn engine.

After identification of isolated FLA as *Allovahlkampfia spelaea*, 3 pair of specific primers were designed using NCBI/Primer-BLAST using *A*. *spelaea* strain SK1 [GenBank:EU696948] as template (shown in Table 1). Amplification and sequencing were done as previously described.

**Table 1:** Primers designed to specifically amplify and sequence 18S ribosomal RNA gene (position is in relation to *Allovahlkampfia spelaea* SK1strain sequence).

	Primer sequence	Start position	Stop position
First	F 5'CGACTGGTTGATCCTGCCAGTAG	1	23
pair	R 5'CATAAGGCCTTTACCAATGGCG	950	929
Second	F 5'CGCCATTGGTAAAGGCCTTATG	929	950
pair	R 5'CTCATACGAACCACCCCGAA	1650	1631
Third	F 5'TTTCTTTCGGGGTGGTTCGT	1626	1645
pair	R 5'TCCTGATCCCTCCGCAGG	2138	2121

Phylogenetic trees were created using Mega6 platform [23], choosing MUSCLE [24] for multiple alignment, and maximum likelihood tree using Tamura-Nei model for generation of tree with gaps removal. Sequences for other amoebae were downloaded from GenBank [accession numbers for 18S ribosomal RNA gene, GenBank: JQ271723, M98052, AJ224887, M18732, FJ169185, GU230754, U94740, AF251938, EU696948, AY425009, AY029409, DQ388520 and for ITS, GenBank: KC820644, AB330071, AJ698838, FJ169186, AJ698839, V00003, AJ132032, K00471, EU696949, KF547910].

### Pathogenicity to animals

In order to confirm the ability of *A*. *spelaea* to produce keratitis, cysts were collected and treated as previously described in axenic trials then suspended in sterile Page’s saline. They were allowed to excyst in culture flask and supernatant was discarded and replaced. The flask was chilled on ice for 3 minutes and shaken to collect trophozoite. Counting was done using hemocytometer and volume was adjusted to have 1x10^5^ trophozoites/ml. Three laboratory rabbits weighing 1500–2000 gms about 3 months old were used for induction of keratitis. Each rabbit was anesthetized using ether, the left cornea was scratched using 27 gauge sterile needle and 10 μl were instilled in its eye. It was kept under anesthesia for 30 minutes to allow adherence of trophozoites. Right eye was only scratched with 27gauge sterile needle. Eyes were examined for the presence of keratitis grossly and with slit lamp.

### Ethics statement

Corneal scrapings were obtained as routine investigation from patient with chronic keratitis. Patient made written consent for using his samples for both diagnostic and research purposes. Faculty of Medicine research ethics committee, Assiut University, approved this study. Animal experiments were done in Animal House, Faculty of Medicine, Assiut University. Animal House ethical committee, Faculty of Medicine, Assiut University, approved them. Animal handling protocols meet the standard international guidelines by the National Institutes of Health guide for the care and use of Laboratory animals and guideline used in other Egyptian universities and research centers.

## Results and Discussion

**Fig 1:**
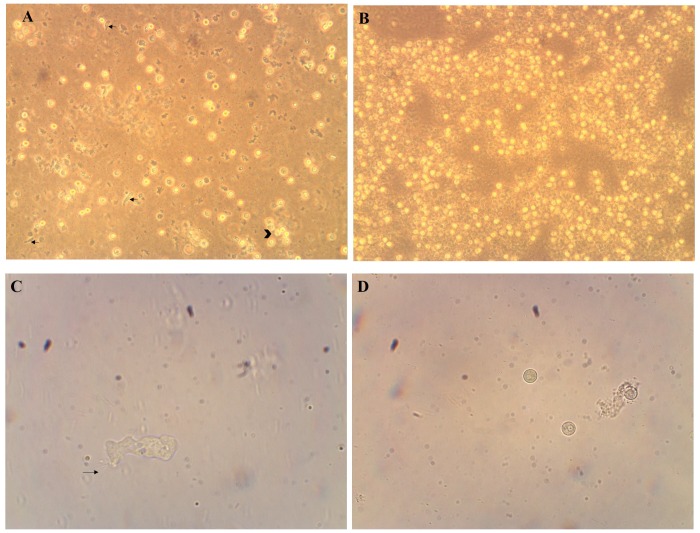
Morphological characters of trophozoite and cyst. (A) Trophozoite showing unidirectional movement (arrows) and cyst aggregating to each other (arrow head) (inverted microscope using x20 objective lens). (B) Cysts in 10 days old culture (inverted microscope using x20 objective lens). (C) Trophozoite showing filopodia (arrow) (oil immersion x100 objective lens). (D) Cysts with perinuclear clear ring (x40 objective lens)

A case of a middle-aged patient with history of trauma to eye and resistant keratitis showed amoeboid trophozoites, which did not resemble *Acanthamoeba* morphologically, on third day of culture. They were pleomorphic with single vesicular nucleus. Movement tends to be unidirectional but not always. Cysts are round with single cell wall and single nucleus with clear perinuclear ring. They tend to aggregate in groups. Once cysts were moved to water, they rapidly excysted and some of the emerging trophozoites showed filopodia (Fig 1). These morphological characters are similar of vahlkamphid amoeba as previously described by Smirnov and Goodkov (1999), Pélandakis and Pernin (2002) Smirnov and Brown (2004), Walochnik and Mulec (2009) and González-Robles et.al. (2012) [3,6,7,15,19]. Trials to grow isolated FLA on TSY medium axenically were done with no success to continuously maintain them.

**Fig 2:**
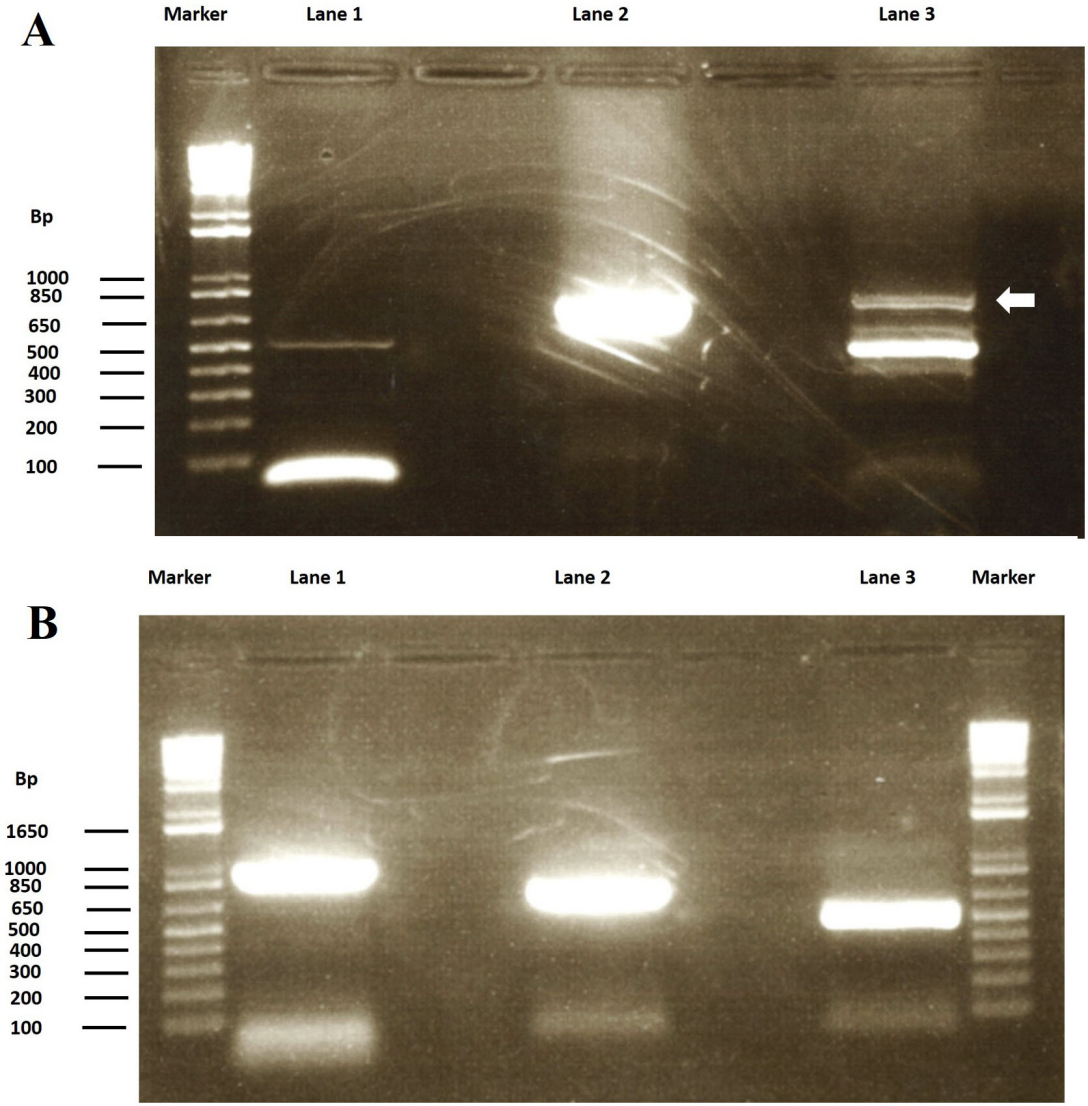
PCR amplification of 18S ribosomal RNA gene. (A) Lane (1) shows faint band of JDP1 & JDP2 primers, lane (2) shows band amplified using short universal primers, while lane (3) shows band specific for 5.8S ITS with auxiliary band (arrow) indicating long variant. (B) Amplification of whole 18S gene using 3 pairs of primers, bands appear at expected molecular weight calculated during primer design.

JDP1 and JDP2 primers were used as standard diagnostic primers for *Acanthamoeba*, but we got a faint band at expected band size (about 515bp). As the morphology of isolated FLA was similar to vahlkamphid amoebae, we tried another 2 pairs of primers. We had good amplification band at universal primers F-566 and R-1200 primers (about 750 bp) and a main 550bp band for Naeg-F and Naeg-R primers with auxiliary faint band (about 750bp) (Fig 2A). Such auxiliary band is indicative of long ITS variant gene[3].

Bands except auxiliary band for ITS gene were excised from gel. DNA was purified and sequenced except for JDP1and JDP2 faint band where another run of amplification was used before sequencing to increase DNA yield. Sequence obtained from JDP1and JDP2 was of low quality indicating non-specific amplification and blast result showed query cover of 59% and identity of 93% for with E value 0 with *Alcaligenes faecalis* strain ZD02 (CP013119.1), a free-living gram-negative bacteria that could co-exist with FLA amoebae. JDP1and JDP2 are used as specific primers for *Acanthamoeba* as suggested by Schroeder et.al. (2001) [16]. This finding confirms that no co-infection with *Acanthamoeba* occurred as the obtained sequence showed no match. It also should raise awareness that some cases of claimed *Acanthamoeba* keratitis diagnosed by PCR technique only could be due to other FLA. The use of JDP1and JDP2 primers is common in clinical practice to diagnose amoebic keratitis without culture. The appearance of such faint band could occur due to binding of primers to non-target sequence leading to weak signal; this is confirmed by sequencing results that showed no significant matches.

Blasting the sequence we had using universal primers F-566 and R-1200 for highly similar sequences, the sequence matched mainly *Allovahlkampfia* with a query cover of 96% and identity of 96% for *A*. *spelaea* strain SK1 and 71–96% for other strain of *Allovahlkampfia* (S1 Fig). Also, blasting the sequence we had using Naeg-F and Naeg-R primers main band, the sequence matched mainly *Allovahlkampfia* with a query cover of 95% and identity of 87% for *A*. *spelaea* strain SK1 with E value 7e-141 and 71–95% for other strain of *Allovahlkampfia* (S2 Fig).

Using the sequence of *A*. *spelaea* strain SK1 18S ribosomal RNA gene (accession number EU696948), we designed 3 pairs of primers to amplify the whole length of 18S ribosomal gene. Amplification was done successfully and DNA was extracted and sequenced (Fig 2B). Resulting sequences were merged using EMBOSS merger (http://emboss.bioinformatics.nl/cgi-bin/emboss/merger) and it was blasted. The merged sequence matched that of *A*. *spelaea* strain SK1 with a query cover of 97% and identity of 97% with E value of zero. The sequence showed only matching to members of family *Vahlkampfidae*. *A*. *spelaea* were first described in 2009 as FLA of caves[19]. This the first time to describe this FLA in Egypt and it is the first time to report it as human pathogen.

**Fig 3:**
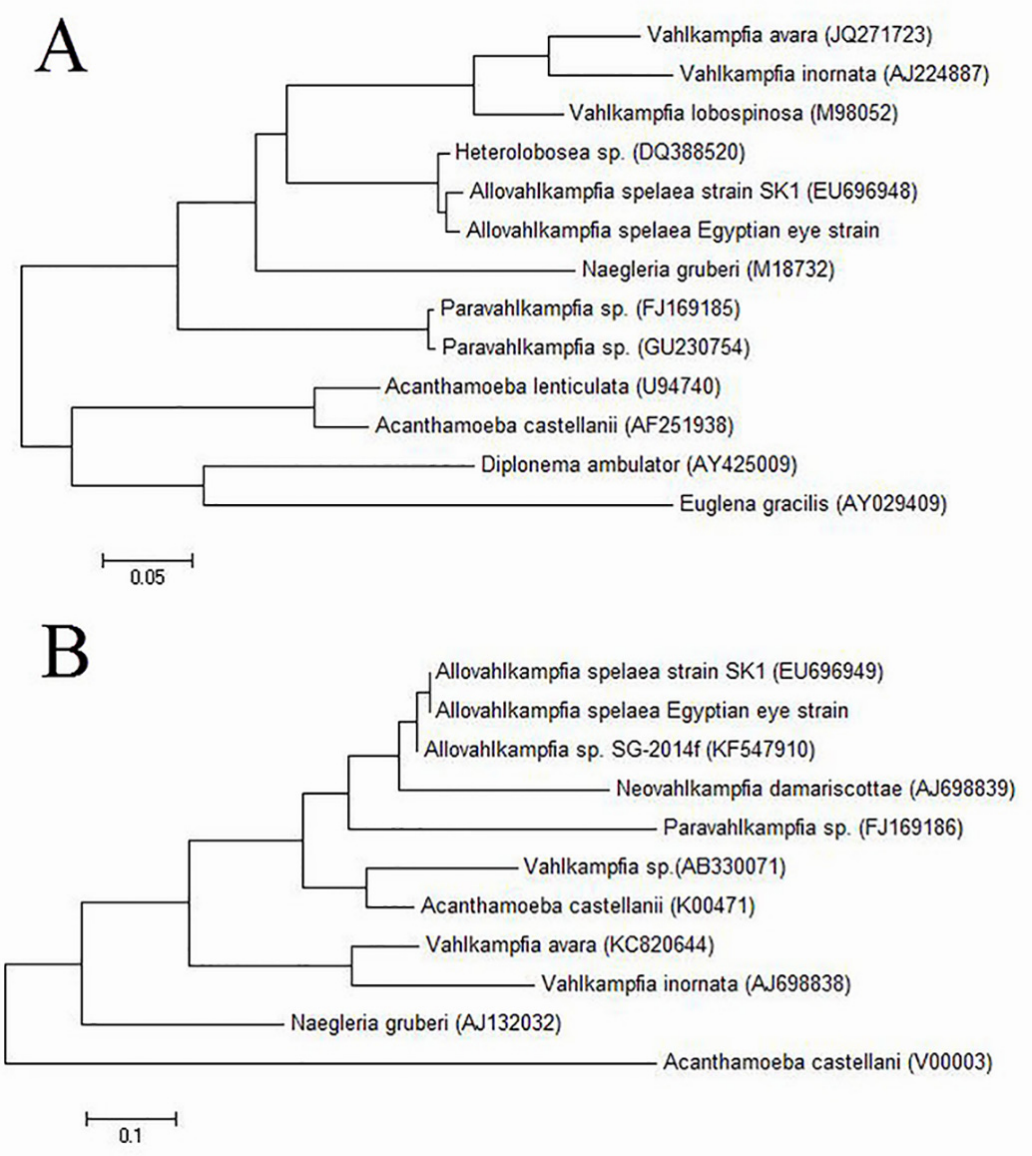
Phylogenetic tree of *Allovahlkampfia spelaea* Egyptian eye strain in relation to other free living amoebae. (A) Tree created using whole length assembled sequence showing close relation to SK1 strain. (B) Tree created using 5.8S ITS sequence showing close relation to SK1 strain.

Using sequences for ITS and complete 18S ribosomal RNA sequence to create maximum likelihood phylogenetic trees showed that the Egyptian eye strain is closely related to *A*. *spelaea* strain SK1 (Fig 3). This indicates that *A*. *spelaea* can be more widely distributed in the world as only limited differences are present between SK1 strain and our strain. All sequences can be found in S1–S9 Files.

**Fig 4:**
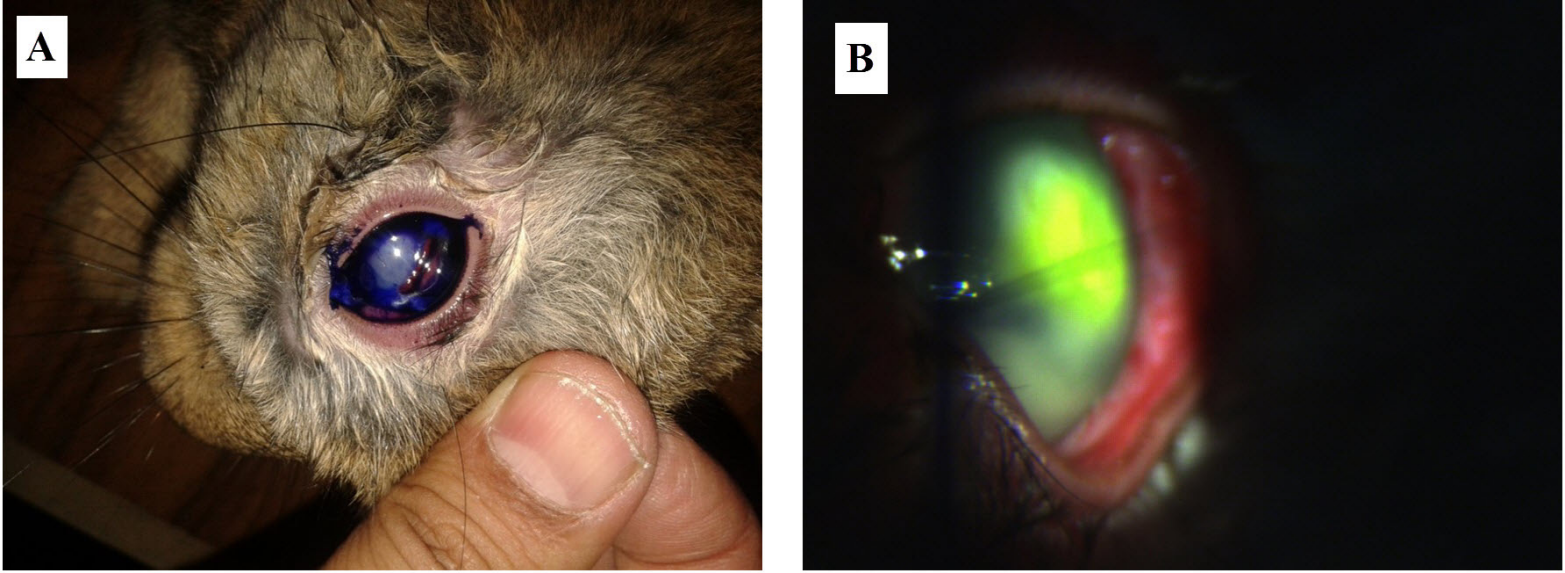
*Allovahlkampfia spelaea* caused definite ulcer to rabbit eye (5 days pi). (A) Rabbit’s eye stained with methylene blue showing ulcer. (B) Rabbit’s eye examined with slit lamp after fluorescein staining to show ulcer (conjunctiva show mild congestion).

To evaluate the pathogenicity of *A*. *spelaea*, rabbit model was chosen as the size of the rabbit’s eye allows easier detection of lesions and easier examination with slit lamp. After 24 hours post inoculation (pi), right eyes of rabbits showed normal cornea while left eyes showed mild keratitis with redness of conjunctiva. On the 3^rd^ day pi, evident corneal ulcers were detected using battery light with or without staining with methylene blue or fluorescein. Rabbits were irritable once light focused on their head. On 5^th^ day pi, ulcer became very large and the degree of conjunctival congestion was not proportional to the size of the ulcer (Fig 4). On 7^th^ day pi, ulcer destroyed most of corneal epithelium in two rabbits and one rabbit showed total loss of epithelium leaving only the basal membrane. These findings prove that *A*. *spelaea* Egyptian eye strain is capable of inducing keratitis in contrary to what was previously speculated with closely related *Vahlkampfia sp*. in which keratitis was thought to be due to mixed infection with *Acanthamoeba*, *Hartmannella* or *Candida*[11,14,25].

Further evaluation is needed to judge the ability of *Allovahlkampfia sp*. to produce keratitis in totally healthy cornea with no history of trauma or friction keratitis as Demirci *et*.*al*. (2006) reported *Acanthamoeba* keratitis in a 5 years old child with no history of trauma or contact lens wearing[10]. The corneal changes appear to be similar to what happens in human case of *Acanthamoeba* keratitis where early in infection only herpetic-like lesions appear which progress in few days to larger ulcer.[10]

### Conclusions

*Allovahlkampfia spelaea* can cause keratitis in humans. This is the 1^st^ time to report such parasite as a human parasite. The presence of FLA in coexistence with each other and with bacteria and fungi makes it necessary to combine both culture and molecular methods for correct diagnosis. For correct molecular diagnosis, use of different primers and sequencing of amplified DNA are important for correct identification of parasite. The close genetic relation between strain isolated in Slovenia and Egypt suggests that the genome of *Allovahlkampfia spelaea* is not much evolutionary separated but further analysis using full genome sequence is needed.

## Supporting Information

S1 Fig Nucleotide Blast result of sequences amplified using short universal primers F-566 and R-1200 showing sequence matches and gaps with *Allovahlkampfia spelaea* strain SK1. (TIF)

S2 Fig Nucleotide Blast result of sequences amplified using Naeg-F and Naeg-R primers to amplify 5.8S ITS gene showing sequence matches and gaps with *Allovahlkampfia spelaea* strain SK1. (TIF)

S1 File Sequence obtained using primers JDP1 and JDP2. (TXT)

S2 File Sequence obtained using primers short universal primers F-566 and R-1200. (TXT)

S3 File Sequence obtained using Naeg-F and Naeg-R primers to amplify 5.8S ITS gene. (TXT)

S4 File Sequence obtained using forward primer of first pair designed to amplify whole 18S ribosomal RNA gene. (TXT)

S5 File Sequence obtained using reverse primer of first pair designed to amplify whole 18S ribosomal RNA gene (reverse-complement sequence). (TXT)

S6 File Sequence obtained using forward primer of second pair designed to amplify whole 18S ribosomal RNA gene. (TXT)

S7 File Sequence obtained using reverse primer of second pair designed to amplify whole 18S ribosomal RNA gene (reverse-complement sequence). (TXT)

S8 File Sequence obtained using forward primer of third pair designed to amplify whole 18S ribosomal RNA gene. (TXT)

S9 File Sequence obtained using reverse primer of third pair designed to amplify whole 18S ribosomal RNA gene (reverse-complement sequence). (TXT)

S10 File Full sequence *Allovahlkampfia spelaea* Egyptian eye-strain 18S ribosomal RNA gene (combined and edited). (TXT)
