# Supplementary figures and images for: Allovahlkampfia spelaea Causing Keratitis in Humans
Source: PLoS Negl Trop Dis. 2016 Jul 14;10(7):e0004841. doi: 10.1371/journal.pntd.0004841 (PMC4945048; doi:10.1371/journal.pntd.0004841)

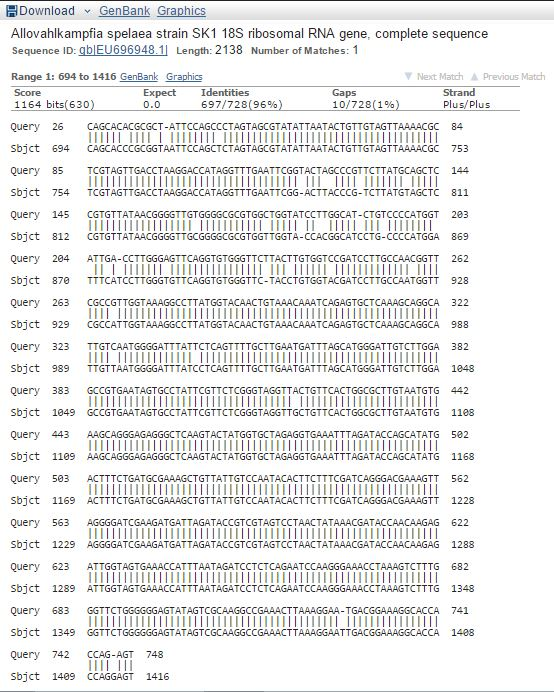

Supplement: S1 Fig — (TIF) [file pntd.0004841.s001.tif]

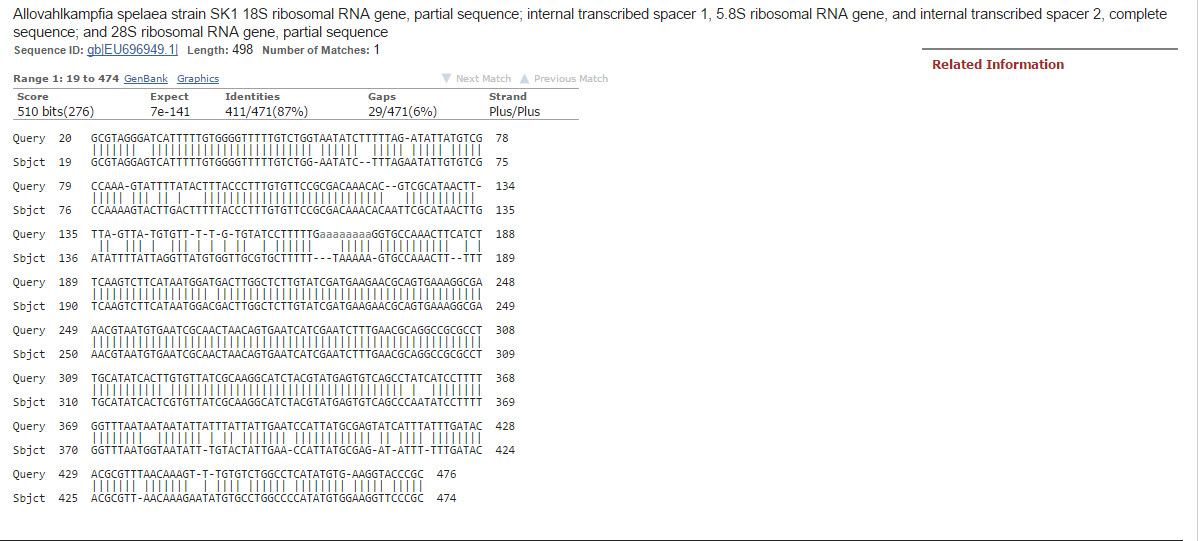

Supplement: S2 Fig — (TIF) [file pntd.0004841.s002.tif]
